# Editorial: Marine Microbial-Derived Molecules and Their Potential Medical and Cosmetic Applications

**DOI:** 10.3389/fmicb.2021.706152

**Published:** 2021-07-16

**Authors:** Jinwei Zhang, Runying Zeng, Antje Labes

**Affiliations:** ^1^Hatherly Laboratories, Institute of Biomedical and Clinical Sciences, University of Exeter Medical School, Exeter, United Kingdom; ^2^European Centre for Environment and Human Health, Environment and Sustainability Institute, University of Exeter Medical School, Penryn, United Kingdom; ^3^Engineering Innovation Center for the Development and Utilization of Marine Bioresources, Third Institute of Oceanography, Minstry of Natural Resources China, Xiamen, China; ^4^Department of Energy and Biotechnology, Flensburg University of Applied Sciences, Flensburg, Germany

**Keywords:** marine microbial ecology, marine biotechnology, bioactive materials, secondary metabolites, enzymes, oligosaccharides, medical and cosmetic applications

The marine environment is a vast, largely unexploited resource for acquiring a multitude of microbial communities with a number of uncharacterized taxonomic groups and novel biosynthetic capabilities. Marine habitats provide unique conditions for microbial growth and secondary metabolite expressions that are not found in terrestrial ecosystems. The co-evolution of many marine macroorganisms, especially invertebrates, with these microorganisms often leads to a very close association or symbiotic relationship between the host organism and specific microbial species driven by natural products. The ocean basin floor is covered in sediments of different types and origins. Marine sediments are considered to be an incredibly rich source of microbial taxonomic diversity, including “unculturable” microbes. “Unculturable” microbial diversity presents a vast gene pool for biotechnological exploitation. In this context the secondary metabolites produced by the microbial species and their biosynthetic pathways represent a resource in the discovery of novel molecules or enzymes for a wide number of applications especially in the medical and cosmetic field. Sustainable use of these resources is a methodological challenge for the exploitation of “unculturable” microbes and their novel gene clusters responsible for biosynthetic pathways for new bioactive compounds and enzymes. However, new culture techniques in addition to culture-independent methods, such as PCR amplification from microbial community DNA (metagenome) and functional or sequence-based screening of metagenomic DNA libraries are proving useful. In recent years, efforts to characterize marine microbial metagenomics and their associated compounds and enzymes have expanded significantly, but given the vastness of the marine environment and the different types of microbial habitats found there, there is still room for game-changing discoveries.

The scope of this Special Issue is to provide a broad and updated overview on marine microbial community dynamics, genome sequencing and gene editing technology, marine microbial-derived molecules, and their potential medical and cosmetic applications. The collection includes 13 original research papers from prominent researchers in the field and provides the readers of the journal with recent results in the area of microbial-derived molecules and their potential application in medical and cosmetic fields. Therefore, this Special Issue promotes our understanding of marine-derived biomolecules and their applications.

Emerging infectious diseases and multi-drug resistant human pathogens are becoming a major threat to global health. There is an urgent need for new antibiotics to fight evolving bacterial infections, especially with respect to gram negative pathogens. The marine habitat was shown to be a good source for extremophiles, known to be potential resources for novel bioactive metabolites. Gao et al. isolated 237 acidophilic/aciduric fungi from Thai mangrove sediment in acidic medium, and identified seven genera including *Penicillium, Aspergillus, Talaromyces, Cladosporium, Phoma, Alternaria, Trichoderma*, in four taxonomic orders of phylum *Ascomycota*. Ninety-five percent of these isolates can grow under extremely acidic conditions (pH 2). Many of them showed cytotoxic, antimicrobial, anti-H1N1 activities. The metabolites obtained from a typical aciduric fungus *Pencillium oxalicum* OUCMDZ-5207 were identified as tetrahydroauroglaucin (1), flavoglaucin (2), and auroglaucin (3), among which compound (3) showed strong selective inhibition on A549 cells with IC50 5.67 μM. Cong et al. isolated 1,208 bacterial and fungus strains in 27 genera from Antarctica soil environments, and investigated their physiology, enzyme activity and antifungal activity, respectively. For example, four polyketones: versicone A (1), versicone B (2), 4-methyl-5,6-dihydro-2H-pyran-2-one (3), and (R)-(+)-sydowic acid (4), were found from *Aspergillus sydowii* strain MS19. Compound (1) displayed strong activity against *Candida albicans*, with a MIC value of 3.91 μg/mL. Sarveswariv et al. screened 42 *Actinobacteria* from a mangrove soil sample, and found the biofilm inhibitory potential of *Micromonospora* sp. RMA46 against *Vibrio cholerae*. They found that the ethyl acetate extract of RMA46, mainly 2-methoxy-4-vinyl phenol and hexahydro-3-(phenylmethyl)-pyrrolo [1,2-a] pyrazine-1,4-dione, inhibited the formation of *V. cholerae* biofilms and downregulated the quorum sensing global switches LuxO and HapR. This study highlights *Micromonospora* sp. RMA46 as a potential source of anti-infectives against *V. cholerae*.

The known peptide sublancin and a surfactin–like lipopeptide demonstrate broad antimicrobial activity against Gram positive bacteria and yeasts. *Bacillus* strains are well-known to produce surfactins however Sharma et al. highlight the lipopeptide synthesized by *Bacillus subtilis* strain A5 isolated from marine sediment, as a candidate to inhibit *Candida ssp*. showing synergistic action with fluconazole. The lipopeptide emulgel formulation showed antimicrobial activity *in vitro* and no skin irritation was observed in mice. Rehman et al. reported nine sorbicillinoid derivatives (1–9), including six new compounds, from a marine sponge-derived fungus *Trichoderma reesei* (HN-2016-018). They discovered two novel sorbicillinoids (1–2) with a characteristic naphthalene-trione ring and two rare sorbicillinoids (3–4) possessing a bicycle (3.2.1) lactone skeleton. Compound 3 represented the first reported sorbicillinoid with a propan-2-one side chain. Compound 5 displayed strong cytotoxic activity against A549, MCF-7, and HCT116 cell lines with the IC50 values of 5.1, 9.5, and 13.7 μM, respectively. Paderog et al. reported about the isolation of precursors of anti-cancer anthracycline shunt metabolites, bisanhydoaklavinone and 1-hydroxybisanhydroaklavinone from marine sediment-derived *Streptomyces sp*. strain DSD069 without genetic manipulation. These two substances have antibiotic activity against *S. aureus* at MIC90 of 6.25 and 50 μg/mL, respectively.

Chemical epigenetic or genetic manipulation has been widely applied to microbes to obtain novel secondary metabolites or enhance the production. Wu et al. investigated the marine gorgonian-derived fungus *Aspergillus versicolor* XS-20090066 by chemical epigenetic manipulation with histone deacetylase inhibitor and DNA methyltransferase inhibitor. The discovered two new nucleoside derivatives, kipukasins K (1) and L (2), and one new bisabolane sesquiterpene, aspergillusene E (7). Compounds 1 and 7 exhibited antibacterial activities against *Staphylococcus epidermidis* and *S. aureus* with the MIC values of 8–16 μg/mL. Compound 7 displayed potent anti-larval attachment activity against bryozoan *Bugula neritina* with the EC50 and LC50 values of 6.25 and 25 μg/mL, respectively. Yao et al. used gene knockout method and created a ΔCtiS mutant strain of from a marine-derived fungus *Penicillium steckii* P2648 for biosynthesis of antibacterial compounds 1, 2, and 3 (isoquinoline alkaloids). Compounds 1 and 2 were reported firstly from the natural resource in this study, showed antibacterial activities against *Staphylococcus aureus* and *Enterococcus faecalis* with the MIC values of 16–32 μg/mL. Atmosphere and room-temperature plasma (ARTP) mutagenesis is an easy and fast method to generate the random mutational library in different strains. Liu et al. used iterative ARTP mutagenesis to obtain mutants from *Streptomyces sp*. MG010 and achieved antibacterial activities significantly increased by 75%.

Most carrageenase degradation products are mixtures, which makes it difficult to purify each type of oligosaccharide. Gui et al. cloned and expressed the carrageenase gene Car3206 in *Escherichia coli* cells, based on the genome sequence of an Antarctic *Polaribacter* sp. strain NJDZ03. The purified recombinant Car3206 produces only disaccharides, which can significantly reduce the cost of product purification in industrial production. Chitosanase is a significant chitosan-degrading enzyme forms chitooligosaccharides. Zheng et al. discovered a new member of GH-46 chitosanase (CsnS) from deep-sea bacterium *Serratia* sp. QD07. CsnS was a cold-adapted enzyme with activity of 324 U/mL, and is favorable to the production of (GlcN)2 and (GlcN)3, may have potential applications in the food and pharmaceutical industries.

Alginate oligosaccharides (AOS) showed various biological activities, with great potentials in industrial and biomedical applications. Marine neoagaro-oligosaccharides (NAOS) produced by most marine β-agarases always possess low DPs ( ≤ 6) and limited categories. Qu et al. developed a novel strategy, using truncated recombinant AgaM1 (trAgaM1), that can efficiently produce NAOS especially with various DPs ≥8. trAgaM1 produced NAOS with various DPs (4–12), for examples, neoagarodecaose (NA10), and neoagarododecaose (NA12) at final concentrations of 3.02, and 3.02 g/L, respectively. Jin et al. investigated AOS with varying degrees of polymerization (DP), namely, AOS (DP2), AOS (DP4–6), and AOS (DP8–12), from a deep-sea agarolytic *Flammeovirga pacifica* strain WPAGA1. They found that AOS could enhance hair growth after 30-days treatment on the back skin of mice.

This Special Issue summarizes important findings related to the marine microbial community, diverse bioactive materials, and their potential applications. The issue provide new possibilities for exploration and research on the novelties of marine microbial natural products.

## Author Contributions

JZ conceptualized the Research Topic and was responsible for writing the whole passage. JZ, RZ, and AL were responsible for checking and revision. All authors have read and agreed to the published version of the manuscript.

## Conflict of Interest

The authors declare that the research was conducted in the absence of any commercial or financial relationships that could be construed as a potential conflict of interest.

